# PREPARE: A Stepped-Wedge Cluster-Randomized Trial to Evaluate Whether Risk Stratification Can Reduce Preterm Deliveries Among Patients With Suspected or Confirmed Preterm Preeclampsia

**DOI:** 10.1161/HYPERTENSIONAHA.122.20361

**Published:** 2023-07-11

**Authors:** Leandro De Oliveira, James M. Roberts, Arundhathi Jeyabalan, Kasey Blount, Christopher W. Redman, Lucilla Poston, Paul T. Seed, Lucy C. Chappell, Marcos Augusto Bastos Dias

**Affiliations:** Botucatu Medical School, Obstetrics Department, Botucatu Sao Paulo State University, SP, Brazil (L.D.O.).; Magee-Womens Research Institute Department of Obstetrics and Gynecology, Epidemiology and Clinical and Translational Research, Pittsburgh, PA (J.M.R., A.J., K.B.).; Nuffield Department of Obstetrics and Gynaecology, University of Oxford, United Kingdom (C.W.R.).; Department of Women and Children’s Health, School of Life Course Sciences, King’s College of London, United Kingdom (L.P., P.T.S., L.C.C.).; Fernandes Figueira Institute, Rio de Janeiro, RJ, Brazil (M.A.B.D.).

**Keywords:** hypertension, morbidity, preeclampsia, preterm birth

## Abstract

**BACKGROUND:**

Early delivery in preterm preeclampsia may reduce the risks for the patient, but consequences of prematurity may be substantial for the baby. This trial evaluated whether the implementation of a risk stratification model could safely reduce prematurity.

**METHODS:**

This was a stepped-wedge cluster-randomized trial in seven clusters. Patients presenting with suspected or confirmed preeclampsia between 20^+0^ and 36^+6^ gestational weeks were considered eligible. At the start of the trial, all centers were allocated in the preintervention phase, and patients enrolled in this phase were managed according to local treatment guidance. Subsequently, every 4 months, 1 randomly allocated cluster transitioned to the intervention. Patients enrolled in the intervention phase had sFlt-1 (soluble fms-like tyrosine kinase-1)/PlGF (placental growth factor) ratio and preeclampsia integrated estimate of risk assessments performed. If sFlt-1/PlGF ≤38 and preeclampsia integrated estimate of risk <10%, patients were considered low risk and clinicians received recommendations to defer delivery. If sFlt-1/PlGF >38 and preeclampsia integrated estimate of risk ≥10%, patients were considered not low risk, and clinicians received recommendations to increase surveillance. The primary outcome was the proportion of patients with preterm preeclampsia delivered prematurely out of total deliveries.

**RESULTS:**

Between March 25, 2017 and December 24, 2019, 586 and 563 patients were analyzed in the intervention and usual care groups, respectively. The event rate was 1.09% in the intervention group, and 1.37% in the usual care group. After prespecified adjustments for variation between and within clusters over time, the adjusted risk ratio was 1.45 ([95% CI, 1.04–2.02]; *P*=0.029), indicating a higher risk of preterm deliveries in the intervention group. Post hoc analysis including calculation of risk differences did not show evidence of statistical differences. Abnormal sFlt-1/PlGF was associated with a higher rate of identifying preeclampsia with severe features.

**CONCLUSIONS:**

The introduction of an intervention based on biomarkers and clinical factors for risk stratification did not lead to reductions in preterm deliveries. Further training on the interpretation of disease severity in preeclampsia and the development of additional risk stratification is needed before adoption into clinical practice.

**REGISTRATION:**

URL: https://www.clinicaltrials.gov; Unique identifier: NCT03073317.

**NOVELTY AND RELEVANCE**

**What Is New?**

This is the first study to combine clinical and laboratory information from the fullPIERS algorithm in the care of patients with suspected or confirmed preeclampsia with the sFlt-1 (soluble fms-like tyrosine kinase-1)/PlGF (placental growth factor) ratio.

**What Is Relevant?**

Early delivery in preterm preeclampsia may reduce the risks for the patient, but consequences of prematurity may be substantial for the baby. Developing risk stratification models to support decisions is a challenge in clinical practice. The risk stratification demonstrated in this study did not lead to reduction in preterm deliveries. However, sFlt-1/PlGF ratio assessment may have contributed to identify patients with severe features of preeclampsia.

**Clinical/Pathophysiological Implications?**

It is important to consider different sFlt-1/PlGF ratio and fullPIERS thresholds for maternal and perinatal outcomes, mainly preterm deliveries. Additionally, developing a continuous knowledge transfer program to help care providers to gain confidence with new tests is crucial for implementation.

**See related article, pp 2029-2032**

Preeclampsia is a complex disorder with diverse phenotypes that affects ≈5% of pregnant women worldwide and is a major cause of maternal and perinatal morbidity and mortality.^1^ The pathological features of preeclampsia can compromise either mother or fetus, but prematurity and perinatal death represent the most important adverse perinatal outcomes.^2^ While delivery reduces the risks of adverse outcomes for the mother, there is an uncertain balance of potential risks and benefits for planned delivery or expectant management for the baby. Therefore, clinical care should always involve a balance between maternal health and prematurity risk, as consequences of unnecessary iatrogenic preterm birth may be substantial for the baby, with important long-term sequelae of chronic neurological disability and adult cardiometabolic diseases.^3–6^

Despite known perinatal disadvantages, mainly at gestational ages before 34 weeks of pregnancy, the incidence of preterm birth among patients with preterm preeclampsia is still high. In Brazil, preeclampsia is the indication for almost 18% of all preterm births and nearly half of all iatrogenic preterm deliveries.^7–9^ In high-income countries, indicated delivery for preeclampsia is also an important contributor to preterm birth but accounts for only about 8% of all premature deliveries.^3^

Current nonstandardized methods of risk stratification use clinical and biochemical criteria, but more recently, improved approaches have been described. Identification of which patients can be safely managed expectantly to minimize the effect of iatrogenic deliveries on perinatal outcomes may be beneficial. The preeclampsia integrated estimate of risk (fullPIERS) algorithm uses maternal symptoms, signs, and laboratory tests to identify patients at increased risk for maternal complications related to preeclampsia, predicting adverse maternal outcomes within 48 hours (area under receiver operating curve, 0.88), with a negative predictive value of 98%.^10^ Adverse outcomes only occurred in 1% of patients with a predicted probability lower than 0.025 and in less than 1% of patients with a predicted probability lower than 0.01. External validation of the fullPIERS model for predicting adverse maternal outcomes in pregnancy hypertension in low- and middle-income countries demonstrated increasing numbers of events according to the progressive increase in prediction score, with a 16.6% risk of adverse events when the prediction score was ≥0.1 (or 10%).^11^ A second available method uses the biomarker sFlt-1 (soluble fms-like tyrosine kinase-1)/PlGF (placental growth factor) ratio as a prognostic marker for maternal adverse outcomes and has the potential to identify patients who do not need a delivery. In a prospective, multicenter, observational study of 500 patients with suspected preeclampsia at <37 weeks, a threshold of ≤38 for an sFlt-1/PlGF ratio had a negative predictive value (no diagnosis of preeclampsia, eclampsia, or hemolysis, elevated liver enzymes, low platelets syndrome within 1 week) of 98.9% with sensitivity of 88.2% and specificity of 80%.^12^ Additionally, a predefined secondary analysis of the Prediction of Short-Term Outcome in Pregnant Women with Suspected Preeclampsia Study demonstrated that the likelihood of imminent delivery among patients (with and without preeclampsia) presenting with sFlt-1/PlGF >38 was 2.9× ([95% CI, 2.4–3.4]; *P*<0.001) greater than in patients presenting with sFlt-1/PlGF ratio ≤38.^13^

The objective of the PREPARE (Prematurity Reduction by Preeclampsia Care) study was to test the hypothesis that risk stratification of patients with suspected or confirmed preeclampsia based on objective criteria reduced the proportion of medically indicated preterm deliveries. The intervention combined fullPIERS and sFlt-1/PlGF ratio in the same treatment guidance to advise obstetricians to defer delivery. Patients with fullPIERS <10% and sFlt-1/PlGF ratio ≤38 were considered as low risk for adverse outcomes and suitable for safe expectant management, while those identified as being at higher risk could be offered appropriate surveillance. Based on this premise, we hypothesized that using this approach we would reduce preterm deliveries related to preeclampsia.

## METHODS

### Data Availability

The data that support the findings of this study are available from the corresponding author upon reasonable request.

### Study Design

This was a stepped-wedge, cluster-randomized trial for the implementation of an intervention at the hospital level to evaluate whether this risk stratification model could safely reduce the incidence of preterm deliveries among patients with suspected or confirmed preeclampsia. The trial was conducted in seven tertiary centers (Table S1), geographically dispersed throughout Brazil, and ran for 33 months. The trial protocol has been previously published.^14^

### Participants

Patients presenting with suspected or confirmed preeclampsia between 20^+0^ and 36^+6^ gestational weeks at any of the seven tertiary centers were considered eligible. Patients with comorbidities (eg, chronic hypertension, renal disease, diabetes) were also included. Suspicion of preeclampsia was a decision defined by clinicians at routine or emergency visits. It was mainly based on blood pressure ≥140 mm Hg systolic or 90 mm Hg diastolic and relevant maternal symptoms such as headache, vomiting, and epigastric pain after 20 gestational weeks. Preeclampsia was defined using the American College of Obstetricians and Gynecologists’ definition as blood pressure ≥140 mm Hg systolic or 90 mm Hg diastolic on or after 20 weeks of pregnancy, plus proteinuria: more than 1+ on a qualitative dipstick, or ≥0.3 g/24 h or urine protein/creatinine ≥30 mg/mmol. In the absence of proteinuria, preeclampsia was diagnosed as blood pressure ≥140 mm Hg systolic or 90 mm Hg in the presence of any relevant predefined maternal symptoms, signs (eg, severe hypertension), abnormal maternal laboratory criteria (eg, low platelets).^15^

Patients with multifetal gestations or those presenting with a diagnosis of fetal demise, nonreassuring fetal status necessitating immediate delivery at admission, eclampsia, hemolysis, elevated liver enzymes, low platelets syndrome, renal, cardiac or respiratory failure, coma, active labor, abruption or emergent delivery for other indications were not included in the risk stratification component of the study.

This study was approved by the national central ethical committee on research involving Human Subjects (CAAE: 53092916·4·2008·5411) and by each local ethical committee of the participating centers. The PREPARE trial was a hospital-wide intervention within a cluster trial. Informed consent at an individual participant level for the intervention was not deemed necessary by the ethics committee.

### Randomization and Masking

Randomization was undertaken by the trial statistician through a computer-generated blocking list at the start of the trial. Random ordering was selected to minimize the rank correlation between the historical event rate and the order of implementation. The timing of implementation of the intervention to each center was revealed only 30 days before the implementation phase to prevent changes in practice before the implementation. However, due to delays with the implementation of the test analyzers and the availability of assays, the initiation of the intervention at sites 2 and 3 was delayed. Before completion of enrollment, the Trial Steering Committee approved the analysis of results according to when the intervention actually happened, rather than when it was originally planned.

At the start of the trial period, all centers were in the preintervention phase. All patients presenting with suspected or diagnosed preeclampsia in this phase were managed according to local guidance. There was no standardization of protocols for preeclampsia treatment or delivery indication. Subsequently, every 4 months, 1 randomly allocated center transitioned to the implementation of the intervention. All centers continued the baseline data collection until randomized to implementation. Once each center crossed over to the intervention phase, the intervention was continued as part of routine care until the end of the study. After the last center has crossed over, the trial continued for a final period of 5 months during which all centers received the intervention. The effectiveness of the intervention was measured by comparing the aggregated data of the centers in the prerandomization phase (preintervention) of the trial with those in the postrandomization phase (post-intervention). Routine clinical data were collected for the trial on all participating patients.

### Procedures

Once the center had transitioned to implement the intervention, all patients had sFlt-1/PlGF ratio measurement and fullPIERS assessment performed as part of their routine care, and results were revealed to the clinicians. sFlt-1 and PlGF were measured using the Elecsys Preeclampsia Platform (Roche Diagnostics) and the results were entered into a trial-specific Global Pregnancy Collaboration database.^16^ Risk stratification using fullPIERS was performed directly using a predefined risk scoring system integrated within the specific Global Pregnancy Collaboration database. These results were available within a time period that permitted timely clinical decision-making (with the intention of results being available within 4 hours of blood sampling), permitting stratification into low-risk or not low-risk for adverse outcomes according to combined assessments of sFlt-1/PlGF and fullPIERS.

- If sFlt-1/PlGF ≤38 and fullPIERS <10% risk, patients were considered low risk. The output provided by the platform was: reassuring—low risk for adverse outcomes. The recommendations were that delivery should be delayed unless the clinical condition deteriorated, with testing repeated at least twice weekly with fullPIERS and at least weekly with sFlt-1/PlGF for reassessment,
- If sFlt-1/PlGF>38 and fullPIERS ≥10% risk, patients were considered not low risk. The output provided by the platform was: nonreassuring—high risk for adverse outcomes, with recommendations to increase surveillance based on the World Health Organization guideline for management and delivery, including the need for preventative therapy with antihypertensive therapy, magnesium sulfate for seizure prophylaxis, and corticosteroids for fetal benefits.^17^ There was no standardization of protocols for delivery indications for the intervention phase. These data were recorded as primarily maternal indication, primarily fetal indication or both. As clinical or laboratory test deteriorations can occur unexpectedly in preeclampsia, the adjudication for decisions was made by clinicians.

### Outcomes

#### Primary Outcome

- Proportion of patients with preterm preeclampsia who delivered <37 weeks’ gestation/total deliveries.

#### Secondary Outcomes

- Proportion of patients with preterm preeclampsia who delivered <37 weeks’ gestation/total deliveries for preeclampsia,
- Proportion of patients with preterm preeclampsia who delivered <34 weeks’ gestation/total deliveries for preeclampsia,
- Prolongation of pregnancy.

Additional maternal outcomes investigated included maternal mortality and or severe morbidity, defined as a composite including eclampsia, hemolysis, elevated liver enzymes, low platelets syndrome, pulmonary edema, stroke or coma, and renal dysfunction. Three further maternal outcomes were considered, hepatic dysfunction, thrombocytopenia, and length of maternal hospital stay, but there was insufficient data to include them.

Additional perinatal outcomes included perinatal death (stillbirth after 27^+6^ weeks or early neonatal death ≤7 days of birth), early neonatal death (≤7 days of birth), and late neonatal death (>7—28 days of birth), Apgar score ≤3 at 5 minutes, respiratory distress syndrome, necrotizing enterocolitis, confirmed sepsis, nonlethal seizures or coma, small for gestational age infants (<10th and third centile). The birthweight assessment was based on the Fenton centile.^18^ Length of neonatal hospital stay and length of neonatal unit stay was also assessed.

### Statistical Analysis

We planned the study around seven clusters, each involving 1 center, and therefore 8 steps each of 4 months; and a total trial length of 8×4=32 months. Based on hospital records, we assumed an average of 330 deliveries per month in each center; and 990 per step (allowing for some delay in the full implementation of the new treatment regime). We considered a clinically important improvement would be a fall from 2.0% to 1.5% in the overall rate of preterm delivery with preeclampsia. We assumed a cluster coefficient of variation of 0.4, giving an intracluster correlation of 0.0024. Power calculations were performed using the Stata command steppedwedge.^19^

Hospital baseline characteristics of pregnancy outcomes to be reported throughout the data collection period included numbers of patients with preterm preeclampsia, gestational age at delivery of all hospital deliveries, and neonatal inpatient nights in patients with preeclampsia. These characteristics were compared between periods before and after the intervention and were summarized by their means and SDs, medians and interquartile ranges, or numbers and percentages as appropriate. Centers were classified as being exposed to the intervention only after randomization.

The primary goal of the study was to test whether a care pathway targeted at preventing unnecessary delivery of patients with preterm preeclampsia would safely reduce the proportion of women delivered in the centers who delivered prematurely. Important independent variables to consider were the clustering effect (ie, the effect of center), calendar time effect (since the intervention was sequentially rolled out), and intervention exposure for each center at each time point, in addition to adjustment for other characteristics. Both individual and cluster-level covariates to be included in the adjustment were prespecified and included maternal age, parity, preexisting chronic hypertension, or other risk factors for prematurity. Null hypotheses and analyses for secondary outcomes took a similar form to that for the primary outcome, with appropriate link functions in the generalized linear mixed model. Summary treatment effect estimates are adjusted and reported (adjusted risk ratios or adjusted odds ratios [aORs]) along with 95% CIs.

Data analysis was performed in Stata version16 (StataCorp, College Station, TX). Standard methods were used for the descriptive tables. For the main analysis, event rates were separately estimated for each center and each month of the trial as a proportion of eligible participants with suspected or diagnosed preeclampsia. Because the month of delivery was not typically the same as the month of trial entry, deliveries were not linked to trial entry.

Analysis of this data set proved more challenging than expected due to several reasons: (1) an unanticipated bias related to most of the centers with the highest event rates occurring late in the randomization sequence; (2) 2 centers starting the intervention later than planned for operational reasons due to external circumstances beyond our control. As this could be regarded as a random event, the Steering Committee decided to focus on the intervention as actually performed, rather than the intervention as originally planned. This we defined as the intention-to-treat dataset. Further, we established a per-protocol subset of the intention-to-treat dataset, restricted to (1) patients in the Intervention arm with an sFlt-1/PlGF ratio results and (2) patients in the Usual Care arm with no test results.

We initially undertook an analysis as indicated in our published protocol.^14^ However, a statistics methodology article published after the trial had been completed (Thompson et al. 2021) questioned the standard methods for analyzing studies of this type, noting an increased risk of a false positive (type 1) error, particularly if there are small numbers of centers per sequence (as in this trial).^20^ We attempted to implement the suggested revised method of analysis. Unfortunately, these methods failed to converge for our data set, and so produced no useable results. Some 5% of the simulations performed by Thompson likewise failed to converge.

Accordingly, we decided to analyze the data from each center separately. The separate analyses all used binomial regression with the total number of deliveries as the denominator, with a linear effect of time as a covariate and a step change at the point the intervention was introduced. We combined the estimates in a random-effects meta-analysis.^21^ While this was not an ideal method, other alternatives had not succeeded in producing a result. Although we had initially intended to adjust for patient-specific variables as specified in the study protocol (maternal age, parity, preexisting chronic hypertension), this was not possible as we did not have that information on all deliveries (the denominator), and therefore the adjustments are for time and center only.

Additional risk differences are shown using the nonparametric within-period cluster-level analysis with the Monte Carlo permutation tests method described in Thompson (2018), demonstrated as reliable.^22^

As it emerged that sFlt-1/PlGF assays were not universally used outside of the trial population, the data safety and monitoring committee suggested an additional post hoc analysis to investigate the proportion of patients with preterm preeclampsia delivered <37 weeks’ gestation among total patients enrolled. Information about the data safety and monitoring committee is provided in the Supplemental Material.

Results are reported as per CONSORT guidelines.^23^

The trial was registered at ClinicalTrials.gov: NCT03073317.

## RESULTS

Between March 25, 2017 and December 24, 2019, 1250 patients with a singleton gestation presenting with suspected or confirmed preeclampsia between 20^+0^ and 36^+6^ gestational weeks were included in the PREPARE trial at seven tertiary centers dispersed across different areas in Brazil. During the randomization process, 2 centers delayed in initiating the intervention due to difficulties obtaining the laboratory machines and reagents. A total of 604 (48.3%) patients were enrolled in the preintervention phase and were managed according to local treatment guidance (usual care group); of these, 41 (4.8%) patients were lost to follow-up, with 563 (93.2%) patients followed to the primary outcome. Six hundred forty-six (51.7%) patients were enrolled in the intervention phase of the centers and were allocated to the risk stratification (intervention group); of these, 60 (9.3%) patients were lost to follow-up and 42 (6.5%) patients did not receive the risk assessment. In 23 women (3.5%), this was due to maternal or fetal problems at first assessment and 19 (2.9%) due to technical problems (biomarker measures not available), thus 586 (90.7%) patients were considered for analysis of the primary outcome. The trial profile is shown in the Figure.

**Figure.**
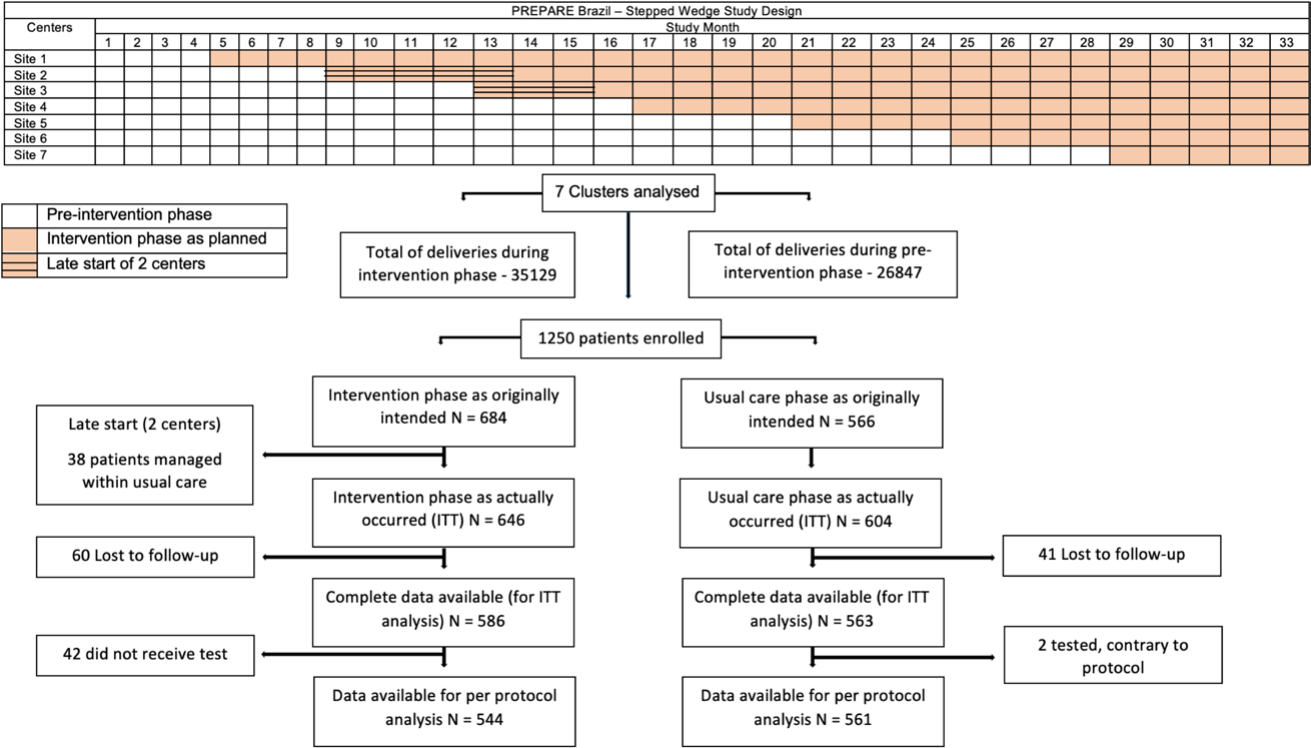
**Description of planned randomization and implementation of the intervention for the stepped-wedge design.** There was an initial 4-month period of baseline data collection at hospitals, during which none of the centers were exposed to the intervention. Subsequently, every 4 months, 1 center was randomized to the intervention. Month 1 started on March 25, 2017; month 33 finished on December 24, 2019. The number of months that each center was observed before and after the implementation of the intervention were site1: 4 and 29 mo; site 2: 13 and 20 mo; site 3: 15 and 18 mo; site 4: 16 and 17 mo; site 5: 20 and 13 mo; site 6: 24 and 9 mo; site 7: 28 and 5 mo.

Maternal demographics and clinical characteristics before enrollment are shown in Table 1. All data were collected from the first antenatal care visit. Different denominators are due to some variables being missing. There were no substantial differences in the baseline characteristics between the 2 trial groups.

**Table 1.**
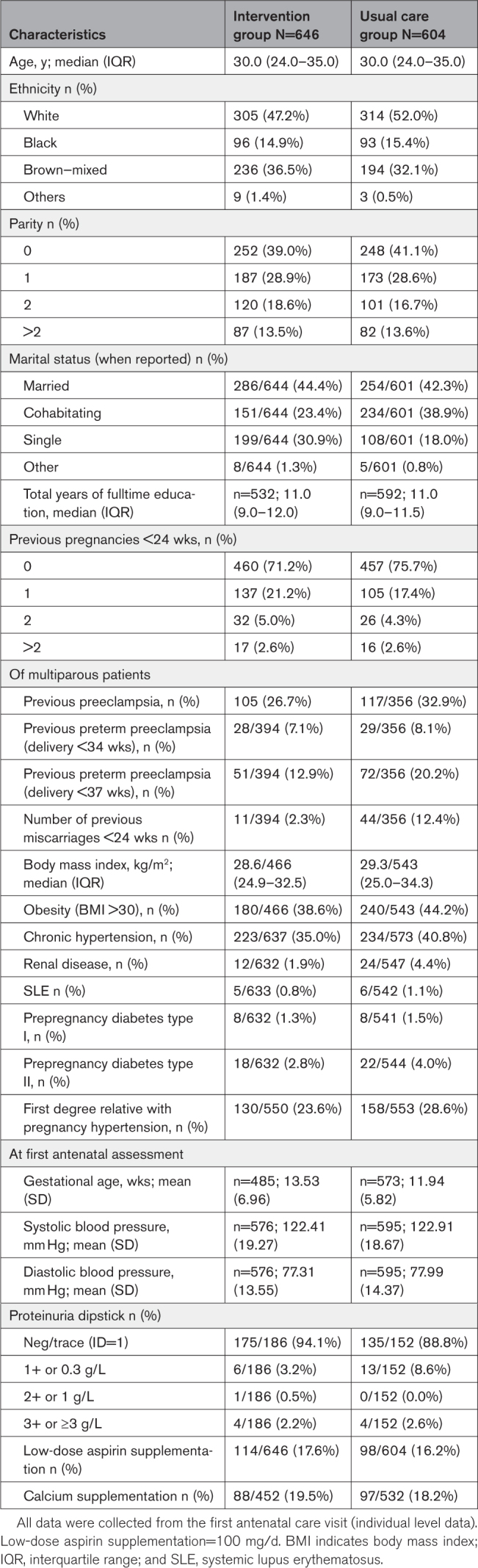
Maternal Demographics and Clinical Characteristics Before Enrollment

Table 2 shows pregnancy characteristics at enrollment. Median (interquartile range) gestational age at first study assessment were 33.7 (30.4–35.7) and 33.0 (30.1–35.1) for intervention and usual care groups, respectively. Additional information on proportions of inclusions according to gestational age is demonstrated in the Supplemental Material (Table S1). Regarding patients enrolled before 24 weeks, only those who reached 24 weeks were considered for analysis. The numbers of patients with suspected preeclampsia or gestational hypertension appeared higher in the intervention group than in the usual care group. There were no substantial differences in presenting symptoms or blood pressure levels at admission. The main antihypertensive agent used in both trial groups was methyldopa, with or without calcium channel blockers. By design, sFlt-1/PlGF ratio and fullPIERS analyses were assessed only in patients enrolled after transition of each center to implementation of the intervention (intervention group). The median (interquartile range) of sFlt-1/PlGF ratio was 67.8 (10.2–256.7); 240 of 594 (40.4%) patients had a ratio ≤38 (reassuring), and 354 of 594 (59.6%) patients had a ratio >38 (nonreassuring) at the time of risk assessment. For fullPIERS results, 550 of 562 (97.8%) patients had a risk evaluation lower than 10% (reassuring) and 12 of 562 (2.1%) presented ≥10% (nonreassuring) at risk assessment. Overall, 357 (60.1%) patients had a nonreassuring sFlt-1/PlGF or fullPIERS test. Therefore, the majority of patients in the intervention group were managed according to recommendations to increase surveillance as advised in the study protocol instead of recommendations to defer delivery (ie, to avoid preterm birth).

**Table 2.**
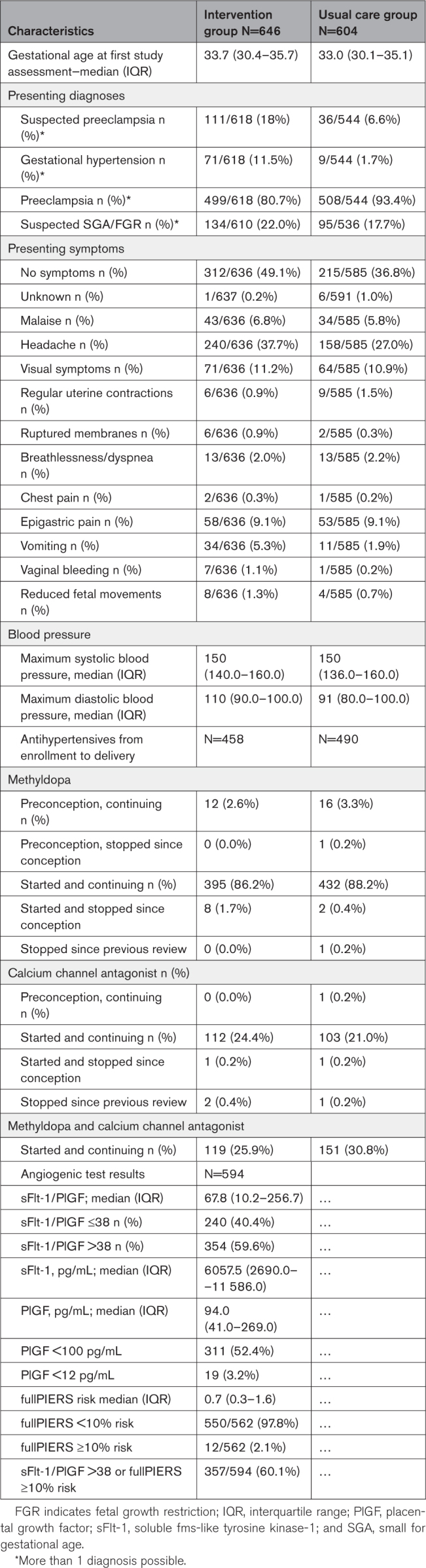
Pregnancy Characteristics at Enrolment

Table 3 shows the main maternal and perinatal outcomes. In the intention-to-treat analysis, there were 375 preterm preeclampsia deliveries out of 35 129 total deliveries in the intervention group compared with 365 preterm preeclampsia deliveries out of 26 684 total deliveries in the usual care group. The number of preterm deliveries and total deliveries by trial center are shown in the Supplemental Material (Tables S2 and S3). The results show an event rate for the primary outcome of 1.09% in the intervention group and 1.37% in the usual care group. However, after prespecified adjustments (primary analysis) for variation between and within clusters over time, the adjusted risk ratio was 1.45 ([95% CI, 1.04–2.02]; *P*=0.029) indicating that there was a higher risk of preterm deliveries in the intervention group. A forest plot of odds ratio in individual clusters and the effect of the intervention on the primary outcome is depicted in the Supplemental Material (Figure S1). This reversal of direction of an effect after adjustments (known as Simpson-Yule paradox) may be explained by the distribution of the event rate by randomized centers, with the event rate occurring ≈4× higher in the 3 last centers randomized as demonstrated in the Supplemental Material (Table S3). Additionally, during a careful analysis of each case in the intervention group, when we examined physician actions on the recommendation based upon a reassuring sFlt-1/PlGF ratio and fullPIERS analysis (last risk assessment) recommending continuing pregnancy, we found that of the 240 patients where reassuring results were recorded, 172 (71.7%) were managed expectantly and 68 (28.3%) were delivered prematurely. Of these 68 patients, 26 (38.2%) were delivered before 14 days, and 42 (61.8%) were delivered after 14 days. Regarding indication for delivery, 2 (2.9%) patients delivered spontaneously, 14 (20.6%) received primarily fetal indication, 39 (57.4%) received primarily maternal indication, and 13 (19.1%) received both indications.

**Table 3.**
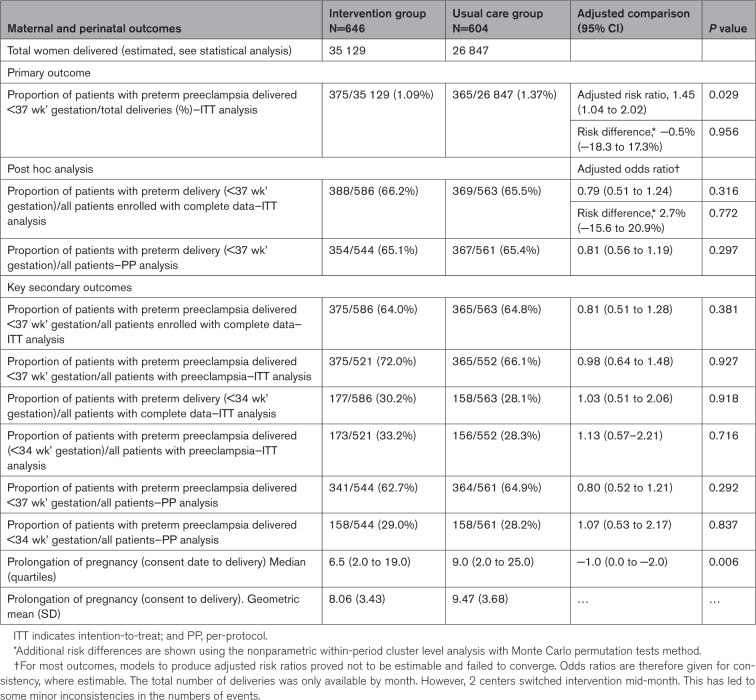
Principal Maternal and Perinatal Outcomes (Tested)

The intention-to-treat post hoc analysis demonstrated that the proportion of patients with preterm delivery (<37 weeks’ gestation) out of all patients enrolled with complete data was 388 of 586 (66.2%) in the intervention group and 369 of 563 (65.5%) in the usual care group; aOR, 0.79 ([95% CI, 0.51–1.24]; 0.316; *P*=0.316) with similar results in the per-protocol analysis (Table 3).

Key secondary outcomes about patients with confirmed preeclampsia are also shown in Table 3. The proportion of patients with preterm preeclampsia delivered <37 weeks’ gestation out of all patients enrolled with complete data was 375 of 586 (64.0%) in the intervention group and 365 of 563 (64.8%) in the usual care group; aOR, 0.81 ([95% CI, 0.56–1.19]; *P*=0.297).

The proportion of patients with preterm preeclampsia delivered <37 weeks’ gestation out of all deliveries for preeclampsia was 375 of 521 (72.0%) in the intervention group and 365 of 552 (66.1%) in the usual care group; aOR, 0.98 (0.64–1.48), *P*=0.927. The proportion of patients with preterm preeclampsia delivered <34 weeks’ gestation out of all deliveries for preeclampsia was 173 of 521 (33.2%) in the intervention group and 156 of 552 (28.3%) in the usual care group; aOR, 1.13 ([95% CI, 0.57–2.21]; *P*=0.716). This denominator was used due to lack of information about total number of preterm deliveries lower than 34 gestational weeks.

The analysis of prolongation of pregnancy (consent date to delivery) demonstrated medians (quartiles) of 6.5 (2.0–19.0) for the intervention group and 9.0 (2.0–25.0) for the usual care group. The aOR was −1.0 ([95% CI, 0.0 to −2.0]; *P*=0.006).

Additional maternal and perinatal outcomes are demonstrated in the Supplemental Material (Table S4). The maternal death reported in the usual care group occurred in an HIV-positive woman who had a stroke (unrelated to the intervention). A composite of events including maternal mortality or severe morbidity (eclampsia, hemolysis, elevated liver enzymes, low platelets syndrome, pulmonary odema, stroke or coma, and renal dysfunction) was evaluated. There was no excess of maternal morbidity in the intervention group, 48 of 586 (8.2%) in the intervention group versus 54 of 563 (9.6%) in usual care group; aOR, 1.14 ([95% CI, 0.59–2.17]; *P*=0.698). Despite no differences in analysis of the composite outcome, after adjustment, the incidence of eclampsia was higher in the usual care group than in the intervention group. There were 7 cases of eclampsia of 583 women (1.2%) in the intervention group and 10 cases of eclampsia of 562 women (1.8%) in the usual care group; aOR, 0.105 ([95% CI, 0.013–0.88]; *P*=0.038). Other additional maternal and perinatal outcomes were similar between the 2 trial groups.

There were no substantial differences in delivery outcomes. Results are demonstrated in the Supplemental Material (Table S5). The mean gestational age at delivery was 34.74 (3.52) weeks in the intervention group and 34.88 (3.40) weeks in the usual care group. Median birthweight was 2323 g (1543–2890) in the intervention group and 2340 g (1555–2918) in the usual care group. Regarding mode of delivery, there were high rates of Caesarean section in both groups. The proportions of Caesarean sections for fetal and maternal indications are shown in the Supplemental Material (Table S3).

The performances of sFlt-1/PlGF ratio and fullPIERS assessments to identify patients with high risk for preterm delivery (primary outcome) and maternal mortality or severe morbidity are demonstrated in the Supplemental Material (Table S6). sFlt-1/PlGF ratio presented good sensitivity to identify patients at high risk for preterm delivery and maternal mortality or severe morbidity. The test performed better than fullPIERS, which had very low sensitivity, but high specificity (based on small numbers with a high-risk result). A secondary analysis of preterm birth about proportion of patients that delivered prematurely among patients with sFlt-1/PlGF ratios ≤38, 38 to 85, and >85 was performed in additional sensitivity analysis. The proportion of patients who delivered prematurely among 517 patients who received either sFlt-1/PlGF or fullPIERS analyses were 68 (36.4%) of 187 patients with ratio ≤38, 53 (73.6%) of 72 patients with ratio 38 to 85 and 226 (87.6%) of 258 patients with ratio >85. The sensitivity analysis is demonstrated in the Supplemental Material (Table S7).

## DISCUSSION

The trial aimed to evaluate whether a combination of the sFlt-1/PlGF ratio and the fullPIERS algorithm, by classifying patients with suspected or diagnosed preeclampsia as low risk for adverse outcomes and therefore suitable for expectant management, would safely lead to a reduction in preterm deliveries.

The main findings showed that the introduction of a new methodology for evaluating patients with elevated blood pressure, characterized by a biomarker test incorporating angiogenic and antiangiogenic factors, did not lead to a reduction in preterm birth. Regarding risk assessment, there was a large discrepancy between the 2 models combined (fullPIERS and sFlt-1/PlGF). The vast majority of patients admitted to the proposed intervention were classified as low risk for adverse maternal outcomes according to the fullPIERS criterion alone. However, 59.6% of these patients had sFlt-1/PlGF ratio >38, indicating a nonreassuring situation in the initial risk assessment. The implementation of the new management protocol, by increasing diagnosis and general awareness of preeclampsia among participating clinicians, may have led to earlier consideration of delivery. Regarding the safety, sensitivity analyses showed that this treatment protocol was able to identify patients at risk for preterm birth and maternal mortality or severe morbidity and, therefore, it did not put patients at risk of adverse outcomes. Overall, no differences were observed about the additional maternal and perinatal outcomes evaluated, except for a reduction in cases of eclampsia in the intervention group.

To our knowledge, this is the first study to combine clinical and laboratory information from the fullPIERS algorithm in the care of patients with suspected or confirmed preeclampsia with the sFlt-1/PlGF ratio. The study evaluated management based on biomarkers in public hospitals that serve a low-income population in Brazil. It was a multicenter study that allowed the inclusion of a large number of patients.

The study evaluated an important perinatal outcome, preterm birth, based on the use of biomarkers that were not in regular use by participating clinicians. We determined that management was different among the participating centers, especially in one of the centers (Figure S1). In addition, eligibility was assessed after randomization in an unblinded way, which may have introduced a risk of bias. Therefore, the results need to be interpreted with caution. Patients with either suspected preeclampsia or confirmed disease were included, and recent evidence has shown that the biomarker test may perform differently in these 2 groups; while PlGF-based testing is useful as a diagnostic adjunct in patients with suspected preeclampsia,^24^ test performance for determining need for delivery in seven days is lower in patients with confirmed preeclampsia.^25^ Further evaluation on biomarker test thresholds and appropriate training for clinicians is needed before these tests are used in clinical practice for patients with established preeclampsia. These initiatives may be useful to increase compliance of clinicians with reassuring results. Finally, another limitation that needs to be considered in the analysis of our results is the rate of loss to follow-up that occurred due to problems in the patient referral system in 2 participating centers at the time of the trial.

Finding a balance between reducing maternal adverse outcomes and the impact of prematurity is a difficult task and there is minimal evidence available to adequately guide this practice. The HYPTAT-II study (2016) compared delivery within 24 hours versus expectant management in patients with hypertensive disorders diagnosed between 34 and 37 weeks.^26^ The group studied did not present with severe disease, and immediate delivery was associated with modest benefit to reverse adverse maternal outcomes. However, the incidence of neonatal respiratory distress syndrome was higher in the immediate delivery group than in the expectant monitoring group. The PHOENIX (Planned Early Delivery or Expectant Management for Late Preterm Preeclampsia Trial) Study (2019) compared planned delivery versus expectant management with individual randomization among patients with late preterm preeclampsia from 34 to <37 weeks’ gestation.^27^ The immediate delivery was associated with lower incidence of adverse maternal outcomes but led to more neonatal unit admissions related to prematurity, without an increase in indicators of neonatal morbidity. If in the future we can accurately identify which patients with preeclampsia are most at risk of adverse maternal or perinatal outcomes, then timing of delivery could be tailored to optimize prolongation of gestation. It is also plausible that a different threshold may be needed for the sFlt-1/PlGF ratio for patients with established preeclampsia. Rana et al^28^ demonstrated that the median (interquartile range) sFlt-1/PlGF ratio in preeclamptic patients who experienced any adverse outcome (N=59) presenting at <34 weeks was 226.6 (50.4–547.3). Identification of higher sFlt-1/PlGF ratios than used in our study to inform the decision to delivery may be more effective in reducing prematurity.

Although the absolute occurrence of the primary outcome decreased after intervention, statistical analysis with appropriate adjustment demonstrated that the adjusted risk ratio was higher in the intervention group. There are several possible explanations for this finding: (1) the risk stratification tests are not adequate to determine timing of delivery in patients with suspected or diagnosed preeclampsia (as suggested by other authors)^10–13^; (2) the tests are good and increase the identification of patients with preeclampsia, particularly of severe features of the disease, leading to appropriately higher concerns by clinicians; and (3) the tests are good for risk stratification, but clinicians did not act appropriately on results. To investigate these hypotheses, we developed sensitivity analyses of the biomarkers involved in the study. In our results, sFlt-1/PlGF >38 ratio showed good sensitivity to identify patients at risk for preterm birth and adverse maternal outcomes. Conversely, the fullPIERS algorithm ≥10% had very low sensitivity to identify patients at risk for preterm delivery and adverse maternal outcomes. Interestingly, the combination of sFlt-1/PlGF and fullPIERS considering both as positive tests, substantially decreased the sensitivity either for preterm delivery or adverse maternal outcomes. But the negative predictive value was high, suggesting the potential of sFlt-1/PlGF as a biomarker to rule out the possibility of adverse outcomes. These results were important to ascertain that the treatment protocol did not put patients at greater risk of adverse outcomes with the intervention but rather may increase the identification of preeclamptic patients with severe features of the disease. Additionally, the post hoc analysis did not show differences about preterm delivery. Endorsing our interpretation, the Data Safety Monitoring Committee monitored all adverse outcomes and allowed the continuation of the study.

In conclusion, this trial demonstrate that implementation of the intervention did not decrease preterm birth for preeclampsia in this setting. The majority of patients enrolled in the trial did not meet the criteria for expectant management due to nonreassuring risk stratification. The threshold used (sFlt-1/PlGF <38) may be too low to lead to an impact in prematurity reduction as most patients entered the trial with higher sFlt-1/PlGF ratios. Future research may need to evaluate the impact of using different test thresholds for maternal and perinatal outcomes, mainly preterm deliveries. We encourage development of new trials in low- and middle-income settings, as these areas have the poorest outcomes for patients with preeclampsia and even small improvements in management may lead to great impact. Additionally, we emphasize the necessity of developing a continuous knowledge transfer program to help care providers to gain confidence and trust in reassuring results.

### Perspectives

To our knowledge, this is the first study to combine clinical and laboratory information from the fullPIERS algorithm with the sFlt-1/PlGF ratio in the care of patients with suspected or confirmed preeclampsia. The main findings of our trial showed that the introduction of a new methodology for evaluating patients with hypertension, characterized by a biomarker test incorporating angiogenic and antiangiogenic factors, did not lead to a reduction in preterm birth.

Interestingly, there was a large discrepancy between the 2 models combined (fullPIERS and sFlt-1/PlGF). A high majority (97.8%) of patients enrolled into the trial were classified as low risk for adverse maternal outcomes according to the fullPIERS criterion alone. However, 59.6% of these women had sFlt-1/PlGF ratio >38, indicating a nonreassuring result in the initial risk assessment.

There was increased knowledge mobilization on preeclampsia by clinicians participating in the study, with an abnormal sFlt-1/PlGF ratio leading to a higher possibility of identifying preeclampsia with severe features, with reduction in cases of eclampsia in the intervention group.

Although the potential role of angiogenic imbalance in risk identification, there is no consensus on a specific cutoff to identify patients at higher risk for adverse outcomes or whether a pattern of elevation of the sFlt-1/PlGF ratio could be useful to define timing of delivery to improve either maternal or perinatal outcomes. Equally, there is still no clear consensus on the threshold of the fullPIERS algorithm that best determines need for delivery, particularly at gestational ages <37 weeks.

Therefore, our results will encourage future researches to evaluate the impact of using different test thresholds on maternal and perinatal outcomes, focusing on optimizing timing of delivery in low- and middle-income settings.

## ARTICLE INFORMATION

### Acknowledgments

The authors thank the independent Data Monitoring Committee. The authors thank all collaborators at the participant centers and their obstetricians, nurses and midwives involved in trial enrollment and management of patients. The authors thank the supporters of the study. This trial was supported by grants from Bill & Melinda Gates Foundation (OPP1142172) and Conselho Nacional de Desenvolvimento Científico e Tecnológico—CNPq and the Brazilian Ministry of Health Brazil (401718/2015-8) to M.A.B. Dias. Roche Diagnostics provided the kits for sFlt-1 (soluble fms-like tyrosine kinase-1) and PlGF (placental growth factor) measurement (Elecsys Preeclampsia Platform). The authors thank all patients who participated in the PREPARE trial (Prematurity Reduction by Preeclampsia Care).

### Sources of Funding

Bill & Melinda Gates Foundation (OPP1142172), Conselho Nacional de Desenvolvimento Científico e Tecnológico—CNPq and the Brazilian Ministry of Health Brazil (401718/2015-8), Roche Diagnostics. The funders of the study had no role in study design, data collection, data analysis, data interpretation, or writing of the report.

### Disclosures

A. Jeyabalan declares Grants or contracts from Mirvie, Inc (Miracle of Life Study) to work as Site PI—overseeing sample collection for a sponsored research agreement between UPMC and Mirvie and Royalties or licenses with UpToDate to work as coauthor of 2 topics—Prevention of Preeclampsia and Chronic Hypertension in Pregnancy. K. Blount declares a contribution to salary paid by Bill and Melinda Gates Foundation to Magee-Womens Research Institute. P.T. Seed declares a contribution to salary paid by Bill and Melinda Gates Foundation to KCL.

### Supplemental Material

Supplemental Methods

Participating centers

Procedures

Knowledge transfer program

Data safety and monitoring committee (DMC)

Tables S1–S7

Figure S1

## Supplementary Material



## APPENDIX

PREPARE Trial group: Guilherme de Jesús MD, PhD; Wallace Mendes-Silva, MD; José Guilherme Cecatti, MD, PhD; Maria Laura Costa, MD, PhD; José Paulo Guida, MD, PhD; Lucienne Frayha, MD; Corintio Mariani-Neto, MD, PhD; Marcos Antonio Nogueira Santos, MD; José Geraldo Lopes Ramos, MD, PhD; Sérgio Hofmeister Martins-Costa, MD, PhD; José Carlos Peraçoli, MD, PhD; Roberto Antonio de Araújo Costa, MD, PhD; Francisco Lázaro Pereira de Sousa, MD, PhD
