# Supplementary material for: PREPARE: A Stepped-Wedge Cluster-Randomized Trial to Evaluate Whether Risk Stratification Can Reduce Preterm Deliveries Among Patients With Suspected or Confirmed Preterm Preeclampsia
Source: Hypertension. 2023 Jul 11;80(10):2017–28. doi: 10.1161/HYPERTENSIONAHA.122.20361 (PMC10510842; doi:10.1161/HYPERTENSIONAHA.122.20361)
Supplement: Supplementary file 1 [file hyp-80-2017-s001.docx]

PREPARE: a stepped-wedge cluster-randomised trial to evaluate whether a risk stratification model can reduce preterm deliveries among patients with suspected or confirmed preterm preeclampsia (Supplemental Material)

Leandro De Oliveira, MD, PhD^1^, James M Roberts, MD^2^, Arundhathi Jeyabalan, MD^2^, Kasey Blount^2^, Christopher W Redman, MB^3^, Lucilla Poston, PhD^4^, Paul T Seed, CStat^4^, Lucy C Chappell^*^, MD, PhD^4^, Marcos Augusto Bastos Dias^*^, MD, PhD^5^ on behalf of the PREPARE trial group

1. Botucatu Medical School, Obstetrics Department, Botucatu Sao Paulo State University, Botucatu, SP, Brazil;
2. Magee-Womens Research Institute Department of Obstetrics and Gynecology, Epidemiology and Clinical and Translational Research, Pittsburgh, PA, USA;
3. Nuffield Department of Obstetrics and Gynaecology, University of Oxford, Oxford, UK;
4. Department of Women and Children’s Health, School of Life Course Sciences, King’s College of London, London, UK;
5. Fernandes Figueira Institute, Rio de Janeiro, RJ, Brazil

*Contributed equally

Short title: Risk stratification in preterm preeclampsia

Word count: 8114

Correspondence to:

Prof Leandro De Oliveira, Obstetrics Department, Botucatu Sao Paulo State University, s/n, Av. Prof. Montenegro, Botucatu - SP, 18618-687, Brazil

e-mail: leandro.gustavo@unesp.br

phone: +55 14 3880 1388

Supplemental methods

Participating centers

The participating tertiary centres and respective cities were: Fernandes Figueira Institute, Rio de Janeiro-RJ; Maternity Maria Amélia Buarque de Holanda, Rio de Janeiro-RJ (these two hospitals constituted one centre for the study); Centre for Women’s Health – CAISM, Campinas-SP; Maternity Leila Diniz, Rio de Janeiro-RJ; Maternity Leonor Mendes de Barros, São Paulo-SP; Hospital of Clinics of Porto Alegre-RS; Botucatu Medical School, Botucatu-SP; Hospital Guilherme Álvaro, Santos-SP.

Procedures

The PREPARE intervention guidance did not recommend delivery based on sFlt-1/PlGF >38 and/or fullPIERS ≥10% risk. In these situations, the trial protocol recommended increased surveillance while delivery indications were based on local guidance and WHO criteria, underpinned by regular clinician training at the start of, and during, the study. Previously published research has not advised using different thresholds of sFlt-1/PlGF or fullPIERS at varying gestations under 37 weeks’ gestation.

All demographic and clinical data were entered into a secure Internet based database designed by the Global Pregnancy Collaboration (CoLab) and maintained by MedSciNet^AB^. This Database (COLLECT) was developed by CoLab (Global Pregnancy Collaboration) with MedSciNet and modified as appropriate for PREPARE, conformed to relevant FDA, NIH, and HL7 standards, guidelines, and recommendations (<http://medscinet.com/projects.aspx)>. Data are held on software deployed on servers located in a data center in Stockholm, operated in compliance with ISO27000 and SAS-70 standards.

Knowledge transfer program

A Knowledge transfer (KT) program was established by two principal investigators, LDO and MABD, to allow the implementation of the intervention. This KT was divided in phases and constituted a strategy to improve commitment with recruitment and adherence to the study protocol.

Phase 1: the KT addressed the problem of non-indicated delivery of patients with preterm preeclampsia, its impact on fetal outcome and its magnitude in Brazil. The evidence for the problem was presented to the staff of the randomised center and the study protocol was presented as possible solution to be investigated. This phase was developed as face-to-face discussions during one day. We emphasized that patients with severe preeclampsia should not be enrolled, as the main objective of the study was to identify patients who did not need to be delivered.

Phase 2: an e-learning modules was developed to provide continuous information about the study. One module presented the problem and supporting data and a second module addressed the use of sFlt-1/PlGF and full-PIERS to grade the risks of delayed delivery.

Module access was monitored by site Principal Investigators. The final module tested what had been learned and those who passed by the whole e-learning module were awarded a formal signed certificate of proficiency.

Importantly, the KT program initiated only when the site was randomised to implement the intervention. Seminars and meetings complemented the strategies developed for study implementation.

Data safety and monitoring committee (DMC)

Two research managers (Leandro De Oliveira and Marcos Dias) of the study monitored data completion regularly and informed responsible researchers at each centre regarding problems to be addressed and corrected. All responsible researchers at each centre were also instructed by the managers to report specific and important serious maternal and perinatal adverse events identified in the trial, including maternal death, fetal or neonatal death, eclampsia, HELLP syndrome, stroke and pulmonary oedema requiring ventilation.

All adverse events were monitored by the Data Monitoring Committee. This committee met every four months to review the progress of the trial.

Supplemental tables

Table S1. Proportion of inclusions according to gestational age (9 missing data)

| Gestational weeks | Intervention group | Usual care group |
| --- | --- | --- |
| <24 | 20 | 13 |
| 24 – 33^+6^ | 322 | 342 |
| 34 – 35^+6^ | 293 | 244 |
| 36 – 36^+6^ | 5 | 2 |

Table S2. Cluster Level Data

| Intervention group | Intervention group | Usual care group |
| --- | --- | --- |
| Total number of deliveries | 35136 | 26942 |
| Number of preterm preeclampsia | 586 | 563 |
| Total number of preeclampsia | 1612 | 1270 |
| Total number of preterm deliveries | 2103 | 1293 |

Table S3. Number of preterm deliveries and total deliveries performed by each trial center in randomised order of intervention.

| Sites | Delivery < 37 weeks | All deliveries | Event rate (%) |
| --- | --- | --- | --- |
| Site 1 | 119 | 17356 | 0.69 |
| Site 2 | 127 | 7254 | 1.75 |
| Site 3 | 77 | 9533 | 0.81 |
| Site 4 | 63 | 15713 | 0.40 |
| Site 5 | 88 | 2639 | 3.33 |
| Site 6 | 182 | 6155 | 2.96 |
| Site 7 | 84 | 3326 | 2.53 |

Table S4: Principal maternal and perinatal outcomes (tested)

| Outcomes | Intervention group  N=646 | Usual care group  N=604 | Adjusted comparison  Odds Ratio (95%CI) | *P* |
| --- | --- | --- | --- | --- |
| Additional maternal outcomes | | | | |
| Maternal mortality n (%) | 0 (0.0%) | 1 (0.2%) | - |  |
| Maternal mortality or severe morbidity | 48/586 (8.2%) | 54/563 (9.6%) | 1.14 (0.59 to 2.17) | 0.698 |
| Severe systolic hypertension (≥160mmHg) n (%) | 295/586 (50.3%) | 255/561 (45.5%) | 0.523 (0.24 to 1.14) | 0.103 |
| Severe diastolic hypertension (≥110mmHg) n (%) | 154/586 (26.3%) | 139/561 (24.8%) | 0.78 (0.31 to 1.93) | 0.587 |
| HELLP n (%) | 40/583 (6.9%) | 45/562 (8.0%) | 1.02 (0.51 to 2.03) | 0.955 |
| Pulmonary oedema n (%) | 4/583 (0.69%) | 2/562 (0.36%) | - |  |
| Eclampsia n (%) | 7/583 (1.2%) | 10/562 (1.8%) | 0.105 (0.013 to 0.88) | 0.038 |
| CVA (stroke and/or coma) n (%) | 0/583 (0.0%) | 1/562 (0.2%) | - |  |
| Renal dysfunction n (%) | 5/583 (0.9%) | 9/562 (1.6%) | 0.26 (0.019 to 3.6) | 0.319 |
| Hepatic rupture n (%) | 0/583 (0.0%) | 0/562 (0.0%) | - |  |
| Additional fetal and perinatal outcomes | | | | |
| Stillbirth (Antepartum) n (%) | 23/599 (3.8%) | 13/576 (2.3%) | 1.55 (0.32 to 7.4) | 0.585 |
| Perinatal death n (%) | 56/598 (9.4%) | 45/576 (7.8%) | 0.81 (0.37 to 1.76) | 0.597 |
| Apgar score ≤3 at 5 minutes | 2/575 (0.3%) | 6/550 (1.1%) | 0.17 (0.017 to 1.77) | 0.139 |
| Respiratory distress n (%) | 205/599 (34·2%) | 220/576 (38·2%) | 1.39 (0.88 to 2.20) | 0.156 |
| Necrotizing enterocolitis n (%) | 10/599 (1.7%) | 8/576 (1.4%) | 1.5 (0.29 to 8.0) | 0.627 |
| Confirmed infection ≤48h n (%) | 15/599 (2.5%) | 35/576 (6.1%) | 0.78 (0.27 to 2.23) | 0.645 |
| Confirmed infection >48h n (%) | 43/599 (7.2%) | 56/576 (9.7%) | 1.16 (0.53 to 2.55) | 0.706 |
| Non-lethal seizures or coma | 8/599 (1.3%) | 18/576 (3.1%) | 0.59 (0.13 to 2.73) | 0.496 |
| Birthweight centile <10^th^ Fenton centile n (%) | 125/593 (21.1%) | 105/574 (18.3%) | 1.06 (0.61–1.85) | 0.836 |
| Birthweight centile <3^rd^ Fenton centile n (%) | 45/593 (7.6%) | 29/574 (5.1%) | 0.74 (0.28 to 1.96) | 0.543 |
| Length of neonatal hospital stay (days) Median (IQR) | 5.0 (3.0,16.0) | 5.0 (3.0,16.0) | - |  |
| Length of neonatal hospital stay (days) Geometric Mean (SD) | n=568  7.63 (2.91) | n=549  7.29 (2.95) | 0.96  (0.57 to 1.61) | 0.863 |
| Length of stay in NNU (if admitted) Median (IQR) | 18.0  (9.0,32.0) | 16.0 (7.0,32.0) | - |  |
| Length of stay in NNU (if admitted) Geometric Mean (SD) | n=281  15.75 (2.77) | n=275  13.99 (2.98) | 0.99  (0.56 to 1.76) | 0.984 |

NOTES: Perinatal death: Stillbirth after 27+ 6 weeks or early neonatal death <7 days of birth; IQR: Interquartile Range; NNU: Neonatal Unit; SD: Standard Deviation.

Table S5: Other maternal and perinatal outcomes (descriptive)

| Outcomes | Intervention group  N=646 | Usual care group  N=604 |
| --- | --- | --- |
| Delivery characteristics | | |
| Gestation at delivery (weeks)  Mean (SD) | n=586  34.74 (3.52) | n=563  34.88 (3.40) |
| Preterm delivery (<37 weeks) n (%) | 388 (66.2%) | 369 (65.5%) |
| Preterm delivery (<34 weeks) n (%) | 177 (30.2%) | 158 (28.1%) |
| Birthweight (g) Median (IQR) | 2323 (1543, 2890) | 2340 (1555, 2918) |
| Labour onset | | |
| Spontaneous | 28 (4.8%) | 39/563 (6.9%) |
| PROM with augmentation | 11 (1.9%) | 9/563 (1.6%) |
| Induction | 167 (28.5%) | 147/563 (26.1%) |
| Termination | 3 (0.5%) | 1/563 (0.2%) |
| Caesarean section | 463/585 (79.1%) | 447/563 (79.4%) |
| Prelabour caesarean section | 400/585 (68.4%) | 395/563 (70.2%) |
| Caesarean section in labour | 63/585 (10.8%) | 52/563 (9.2%) |
| Unknown | 1/586 (0.2%) | 1/563 (0.2%) |
| Indication for Caesarean section n (%) | | |
| Primarily fetal problems | 129/463 (27.9%) | 160/447 (35.8%) |
| Primarily maternal problems | 195/463 (42.1%) | 170/447 (38.0%) |
| Both | 78/463 (16.8%) | 74/447 (16.5%) |
| Planned method of delivery | 59/463 (12.7%) | 46/447 (10.3%) |
| Unknown | 2 (0.4%) | 2 (0.4%) |
| Antihypertensive treatment in labour n (%) | 230/556 (41.4%) | 193/509 (37.9%) |
| Hydralazine | 133/158 (84.2%) | 90/129 (69.8%) |
| Nifedipine | 25/158 (15.8%) | 38/129 (29.5%) |
| Use of magnesium sulfate n (%) | | |
| Severe preeclampsia | 290/570 (50.9%) | 261/521 (50.1%) |
| Eclampsia | 7/570 (1.2%) | 10/521 (1.9%) |
| Fetal neuroprotection (preterm delivery) | 31/570 (5.4%) | 17/521 (3.3%) |
| Additional fetal and perinatal outcomes | | |
| Discharged home n (%) | 310/623 (49.8%) | 289/590 (49.0%) |
| Transferred to Neonatal Unit n (%) | 274/623 (44.0%) | 264/590 (44.7%) |
| Antenatal corticosteroids for fetal lung maturation n (%) | 39/623 (6.3%) | 37/590 (6.3%) |
| Apgar score at 5 minutes  Median (IQR) | n=600  9.0 (9.0,10.0) | n=559  9.0 (8.0,10.0) |
| Early neonatal death (<7 days) n (%) | 25/600 (4.2%) | 23/559 (4.1%) |
| Late neonatal death (7-28 days) n (%) | 7/600 (1.2%) | 10/559 (1.8%) |

PROM: Prelabour rupture of membranes; IQR: Interquartile Range.

Table S6. Performance of sFlt-1/PlGF ratio and fullPIERS assessment to identify patients with high-risk for preterm delivery (Primary outcome) and maternal mortality or severe morbidity (517 patients receiving both tests only)

| Preterm delivery (Primary outcome) | | | | |
| --- | --- | --- | --- | --- |
| Test results | sFlt-1/PlGF >38 | fullPIERS ≥10% | Both tests positive  (sFlt-1/PlGF >38 and fullPIERS ≥10%) | At least one test positive (sFlt-1/PlGF >38 or fullPIERS ≥10%) |
| Prevalence of primary outcome % | 67.1  (62.9 to 71.2) | 67.1  (62.9 to 71.2) | 67.1  (62.9 to 71.2) | 67.1  (62.9 to 71.2) |
| Sensitivity % | 80.4  (75.8 to 84.4) | 2.9  (1.4 to 5.2) | 2.3  (1.0 to 4.5) | 81.0  (76.4 to 85.0) |
| Specificity % | 70.0  (62.5 to 76.8) | 100.0  (97.9 to 100.0) | 100.0  (97.9 to 100.0) | 70.0  (62.5 to 76.8) |
| Balanced accuracy | 75.2  (71.2 to 79.2) | 51.4  (50.6 to 52.3) | 51.1  (50.4 to 51.9) | 75.5  (71.5 to 79.5) |
| PPV n/N % | 84.5  (80.2 to 88.3) | 100.0  (69.2 to 100.0) | 100.0  (63.1 to 100.0) | 84.6  (80.3 to 88.3) |
| NPV n/N % | 63.6  (56.3 to 70.5) | 33.5  (29.4 to 37.8) | 33.4  (29.3 to 37.7) | 64.3  (57.0 to 71.2) |
| Likelihood ratio (+) | 2.7  (2.1 to 3.4) | - | - | 2.70  (2.13 to 3.42) |
| Likelihood ratio (-) | 0.7  (0.2 to 0.3) | 1.0  (0.95 to 0.99) | 1.0  (0.96 to 0.99) | 0.27  (0.21 to 0.34) |
| Odds Ratio | 9.6  (6.15 to 14.93) | - (1.30 to -) | - (1.04 to -) | 9.93  (6.37 to 15.53) |
| AUROC | 0.8  (0.78 to 0.85) | 0.6  (0.59 to 0.69) | - |  |
| Maternal mortality or severe morbidity | | | | |
| Test results | sFlt-1/PlGF >38 | fullPIERS ≥10% | Both tests positive  (sFlt-1/PlGF >38 and fullPIERS ≥10%) | At least one test positive (sFlt-1/PlGF >38 or fullPIERS ≥10%) |
| Prevalence of maternal mortality or morbidity n/N % | 7.5  (5.4 to 10.2) | 7.5  (5.4 to 10.2) | 7.5  (5.4 to 10.2) | 7.5  (5.4 to 10.2) |
| Sensitivity % | 87.2  (72.6 to 95.7) | 10.3  (2.9 to 24.2) | 5.1  (0.6 to 17.3) | 92.3  (79.1 to 98.4) |
| Specificity % | 38.1  (33.7 to 42.6) | 98.7  (97.3 to 99.5) | 98.7  (97.3 to 99.5) | 38.1  (33.7 to 42.6) |
| Balanced accuracy | 62.6  (57.0 to 68.3) | 54.50  (49.7 to 59.29) | 51.94  (48.4 to 55.4) | 65.19  (60.48 to 69.91) |
| PPV n/N % | 10.3  (7.2 to 14.1) | 40.0  (12.2 to 73.8) | 25.0  (3.2 to 65.1) | 10.8  (7.7 to 14.7) |
| NPV n/N % | 97.3  (93.9 to 99.1) | 93.1  (90.5 to 95.1) | 92.7  (90.1 to 94.8) | 98.4  (95.3 to 99.7) |
| Likelihood ratio (+) | 1.4  (1.2 to 1.6) | 8.17  (2.4 to 27.7) | 4.09  (0.85 to 19.57) | 1.5  (1.3 to 1.7) |
| Likelihood ratio (-) | 0.3  (0.1 to 0.8) | 0.91  (0.82 to 1.01) | 0.96  (0.89 to 1.03) | 0.20  (0.07 to 0.60) |
| Odds Ratio | 4.2  (1.6 to 13.9) | 9.0  (1.8 to 39.6) | 4.25  (0.40 to 24.75) | 7.4  (2.3 to 37.9) |
| AUROC | 0.69  (0.62 to 0.77) | 0.80  (0.73 to 0.87) | - |  |

NOTES: Numbers in parentheses are 95% Confidence Interval; PPV: Positive Predictive Value; NPV: Negative Predictive Value; AUROC: Area Under Receiver Operating Characteristics; Where ROC Areas cannot be estimated, the balanced accuracy (Mean of sensitivity and specificity) has similar properties.

Table S7. Performance of different sFlt-1/PlGF ratios to identify patients with high-risk for preterm delivery (517 patients receiving both tests sFlt-1/PlGF and fullPIERS only)

| Preterm delivery (Primary outcome) | | |
| --- | --- | --- |
| Test results | sFlt-1/PlGF >38 | sFlt-1/PlGF >85 |
| Prevalence of primary outcome % | 67.1  (62.9 to 71.2) | 67.1  (62.9 to 71.2) |
| Sensitivity % | 80.4  (75.8 to 84.4) | 65.1  (59.9 to 70.1) |
| Specificity % | 70.0  (62.5 to 76.8) | 81.2  (74.5 to 86.8) |
| Balanced accuracy | 75.2  (71.2 to 79.2) | 73.1  (69.29 to 77.02) |
| PPV n/N % | 84.5  (80.2 to 88.3) | 87.6  (82.9 to 91.4) |
| NPV n/N % | 63.6  (56.3 to 70.5) | 53.3  (47.0 to 59.5) |
| Likelihood ratio (+) | 2.7  (2.1 to 3.4) | 3.46  (2.51 to 4.77) |
| Likelihood ratio (-) | 0.7  (0.2 to 0.3) | 0.43  (0.37 to 0.50) |
| Odds Ratio | 9.6  (6.15 to 14.93) | 8.05  (5.08 to 12.9) |

NOTES: Numbers in parentheses are 95% Confidence Interval; PPV: Positive Predictive Value; NPV: Negative Predictive Value; The balanced accuracy (Mean of sensitivity and specificity) has similar properties of ROC curves.


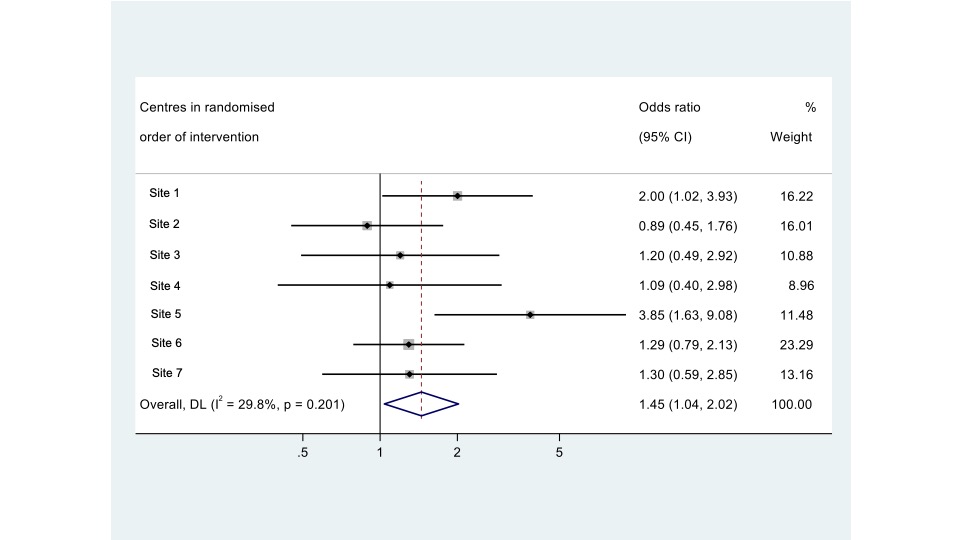


Figure S1. Forest plot of adjusted Odds Ratios in individual clusters and the effect of the intervention on the primary outcome.
